# Anti-Human H1N1pdm09 and swine H1N1 Virus Antibodies among Swine Workers in Guangdong Province, China

**DOI:** 10.1038/srep12507

**Published:** 2015-07-24

**Authors:** Jie Wu, Lina Yi, Hanzhong Ni, Lirong Zou, Hongbin Zhang, Xianqiao Zeng, Lijun Liang, Laiqing Li, Haojie Zhong, Xin Zhang, Jin-yan Lin, Changwen Ke

**Affiliations:** 1 Guangdong Provincial Center for Disease Control and Prevention, Guangzhou, China; 2 Guangdong Provincial Institute of Public Health, China; 3 Guangzhou General Hospital of Guangzhou Military District, China; 4 Guangzhou Youdi Biotech Co., LTD.

## Abstract

To assess the potential transmission for zoonotic influenza, sero-antibodies against two kinds of influenza viruses—classical swine H1N1 and human H1N1pdm09 virus were detected in persons whose profession involved contact with swine in Guangdong province, China. Compared to the non-exposed control group, a significantly higher proportion of subjects with occupational contact to pigs exhibited positive seroreaction against the classical H1N1 SIV. Participants aged 26–50 years were at high risk of classic swine H1N1 infections. Seropositive rate to 2009 pandemic H1N1 virus among swine workers was similar with controls. The major impact of age was apparent for younger populations. Our present study has documented evidence for swine influenza virus infection among persons with occupational swine exposures. The differences of seroreactivity for the two tested influenza subtypes emphasize the necessity of regular surveillance both in pigs and human.

Influenza A virus (FluA) is a highly contagious respiratory virus. It can infect a wide variety of species, including human, pigs, birds and horses^1^. Although being high host specificity, interspecies transmission of FluA following genetic reassortment has occurred occasionally^1^,^2^,^3^. Be susceptible to both human and avian viruses, pigs are recognized as mixing vessels for influenza viruses. Novel influenza viruses with high pathogenicity and transmissibility may emerge in pigs via genetic adaptive mutation or gene reassortment^4^.

Virological and serological surveillance for swine influenza viruses (SIVs) have been performed in China for more than ten years^5^,^6^. It is documented that all main SIV subtypes are recently co-circulating in China^7^. Classical swine (CS) H1N1 is the first identified SIV. Its presence in China probably dates back to 1918-1919, when a disease closely resembling influenza in humans occurred in pigs in Chinese coastal cities^8^. Although the epidemiologic evidence in mainland China before 1990s is fragmentary, continuing presence of CS H1NI in China since the mid-1970s could be inferred from surveillance conducted in Hong Kong, where 80–95% of the swine imported from mainland China^9^. And CS H1N1 virus was the predominant influenza virus isolated before 2002^10^. Evolutionary studies revealed multiple introductions of CS from American pigs into Chinese pigs. In contrast, the European Avian (EA)-like H1N1 viruses, which emerged in Chinese pigs in 2001, are results of a single introduction from Europe and have an entirely avian genome^10^,^11^. The H1N2 swine influenza viruses currently circulating in China was a triple-reassortant swine (TRIG) virus generated in North America in 1998^12^. Since 2002, regular isolation of this virus was reported in China^10^. Another TRIG SIV generated in the reassortment event of 1998 is the TRIG H3N2 swine viruses. This virus along with other three types of H3N2 viruses (wholly human origin, wholly avian origin and double reassortants H3N2) compose main H3N2 influenza viruses discovered in Chinese pigs^13^. Gene bank and serological studies of the SIV showed that CS, EA, TRIG and H3N2 viruses were widely distributed in China during 2002–2005^10^. After that, EA H1N1 viruses became dominant and have co-circulated with CS and TRIG viruses. Then, the outbreak of 2009 pandemic occurred. Genetic characterization shows that this virus is a quadruple reassortant containing genes from classical H1N1 swine influenza virus, human seasonal H3N2 influenza virus, North American avian influenza virus, and Eurasian avian-origin swine influenza viruses^14^. And as the result of human-to-pig transmission, the 2009 pandemic like virus has been repeatedly isolated from pigs. Recent reports show that reassortments between H1N1pdm09 virus and endemic swine viruses have occurred repeatedly^15^, which arouse concerns that the next pandemic virus is likely to be H1N1pdm09 virus origin and arise in swine.

Zoonotic infections with SIVs have been described in many literatures. The environment and lifestyle of swine farm workers make them be the frontline of infecting SIVs^16^. Be routinely exposed to swine, Swine workers, on the one hand, could introduce human influenza viruses into swine populations and increase the probability of viral reassortment; and on the other hand this group of population could mediate the movement of a novel virus with pandemic potential from swine to human. Seroepidemiology studies on swine workers could provide indirect evidence of SIV transmission to humans. Previous study revealed that 11.7% of swine farm residents in Southern China had sero-antibodies against EA H1N1 virus. Occupational exposure may increase their risk of EA SIV infections^17^. In this study, serological antibody levels direct against CS H1N1 and H1N1pdm09 virus were detected for persons whose professions involved contact with swine.

## Methods

### Study Population

In order to determine the level of antibodies against CS H1N1 and H1N1pdm09 viruses in persons whose professions involved contact with swine, a seroepidemiology study was conducted in Guangdong province, Southern China. Swine workers including swine keepers, pork processer and quarantine officials were identified and selected randomly. The stalls in wet market in China are quite crowded. No sufficient space is allotted between different stalls. And retailer in same wet market usually share cleaning water and dump sites. All above mentioned lead to regular exposure to pork for retailers of goods other than pork in wet markets, and thus this group population was also enrolled and classified as exposure group in our study. A total of 712 participants from 17 workplaces (5 slaughter plants, 10 pig farms and 2 food markets) were initially enrolled. As a control, a group of blood donors who had no occupational exposure to pigs were recruited in the study. Serum samples from participants were collected between Apr 2013 and May 2014. Survey questionnaire was completed by trained interviewers and included information on the subject’s age, gender, and the nature of their contact with pigs. All participants had no sign of disease at the time of sample collection. And none had received vaccines against seasonal or H1N1pdm09 virus. Written informed consent was obtained from individual study participants. The study was approved by the ethics committee of the Guangdong Provincial Center for Disease Control and Prevention, and was in compliance with the Helsinki Declaration.

### Viruses and laboratory procedures

For the present study, the following two viruses were used. 1) A pandemic influenza A virus: A/Guangdong/501/2010/H1N1pdm09. This virus was isolated from a nasopharyngeal aspirate of a patient who reported Influenza like Illness by using 10-day-old SPF egg in our laboratory. Sequence analysis revealed almost identity to A/California/07/2009(H1N1). The GenBank accession number is KR030166. 2) A CS H1N1 virus: A/swine/Guangdong/L6/09. This virus was obtained from College of Veterinary Medicine, South China Agricultural University. This virus was isolated from a dyspneic pig of Guangdong in March 2009. And it belongs to the lineage of classical swine influenza virus and all of eight segments are swine origin. The GenBank accession number is HQ880611–HQ880618. Viruses were propagated in embryonated chicken eggs and inactivated with β-propiolactone (Sigma, St. Louis, MO). Amino acid sequence comparisons between haemagglutinin proteins (HA) of these two viruses were conducted in MEGA5.0 (Molecular Evolutionary Genetics Analysis software version 5.0).

The haemagglutination inhibition (HI) assay was performed as described previously and in accordance with the WHO recommendations^18^,^19^. Serum samples were pretreated with Receptor destroying enzyme (RDE) (DENKA SEIKEN Ltd, Japan) for 19 hours at 37 °C to remove non-specific serum inhibitors and inactivated at 56 °Cfor 30 minutes. For the HI assay, two fold serial dilutions of serum samples were added in V-shaped micro plate and four hemagglutinating units of viruses were added to each well. The mixture was incubated at room temperature for 35 min. Then, 0.5% (v/v) horse erythrocytes were added to each well. The plates were left at room temperature for 40 min. HI assay with two fold serial dilutions starting from 1:10 was duplicated per virus. The HI titers were expressed as the highest dilution of serum giving complete inhibition of agglutination. And HI titers ≥1:40 were defined as serological positive. To examine potential cross-reactivity, HI titers from control antisera were determined against the reference virus strains (e.g., antisera to CS H1N1 virus was examined against human H1N1pdm09 virus).

Neutralization test (NT) was conducted for partial randomly selected serum samples in this study. 10 μl of heat-inactivated sera including positive serum, negative serum and serum samples were added to 96 well cell culture plate (Nunc Corp) and performed 2-fold serial dilutions. 100TCID50/50 μl virus was added to wells and the virus-serum mixture was incubated for 1 hour at 37 °C, 5% CO2. Back-titration, started with the virus test dilution (100 TCID50) and a prepared additional 2-fold serial dilution with diluents was set-up. 100 μl MDCK cells (1.5 × 10^4^ cells/well) were then added to each well of plate and incubated overnight at 37 °C, 5% CO2 (18–20 hours). The plate was fixed with 100 μl/well of cold fixative at RT for 10 min. Then the virus was detected with anti-NP monoclonal antibody (KPL Company) and HRP-goat anti-mouse IgG (SANTA CRUZE) as secondary antibody by using ELISA. The value below X(X = (Average OD of Positive cell control wells-Average OD of negative cell control wells)/2+ (Average OD of negative cell control wells)) was positive for neutralization activity.

### Statistical analysis

Statistical analyses were performed with SPSS version 13.0. The proportions of seropositive participants for each age group, exposed status (exposed and control), work type and sex were calculated according to the binomial distribution. Bivariate and unconditional logistic regression were used to examine risk factors associated with seropositivity to CS H1N1 and H1N1pdm09 viruses, as described previously^20^. The variables with P < 0.10 in bivariate analysis were included in unconditional logistic regression model. Enter logistic regression was conducted. Crude odds ratio (OR) and adjusted odds ratio with 95% confidence intervals (95% CI) were calculated. Statistical significance was established assuming an alpha error of 0.05. HI titers of positive serum samples were log-transformed to calculate the geometric mean titer (GMT) and 95% confidence intervals (CI). Kruskal–Wallis and Mann–WhitneyU tests were used for comparison of the GMT among different groups. Antibody titers <1:40 were assigned a value of 20.

## Results

### Characteristics of the subjects in the study

A total of 1214 subjects were recruited in the study. The demographic profiles of participants are presented in Table 1. The exposed group consisted of 712 participants from four types of occupations. Of these, 126 (17.7%) were swine keepers, 169 (23.7%) were pork processer (including pig butchers and pork retailers), 360 (50.6%) were retailers of goods other than pork in food market and 57 (8.0%) were quarantine officials. For control, 502 blood donors who had no occupational exposure to pigs were enrolled. The mean age was 39.7 years old (SD 12.4) in the exposed and 32.3 years old (SD 16.8) in the unexposed subjects. The sex ratios of male to female were 1.67:1 and 1.47:1 respectively. According to the questionnaire, none of the participants had received influenza vaccination.

### Antigenic characteristics of viruses

**Figure 1:**
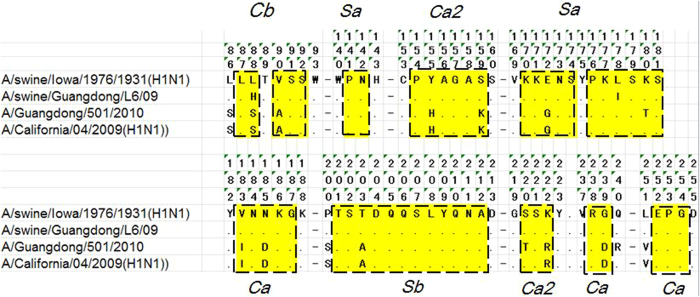
Amino acid sequence comparisons of epitopes of HA among the detected antigens and their references.

Amino acid sequence comparisons between HA proteins of these two viruses were conducted with A/California/07/2009(H1N1) as human reference and A/Swine/Iowa/1976/1931(H1N1) as swine reference (Fig. 1). Comparison between the two tested viruses revealed 82% identity for HA amino acids. Of the 49 residues located at five antigenic sites, the tested A/Guangdong/501/2010/H1N1pdm09 and A/swine/Guangdong/L6/09 differed at thirteen amino acid sites (H88S, V90A, Y155H, S159K, E172G, I178L, K180T, V183I, N185D, T203A, S220T, K222R, and G239D). Meanwhile, only two amino acids were different between the tested antigens and the control antigens (L88H and L178I for CS H1N1, T180K and T220S for H1N1pdm09 virus).

Serologic cross-reactivity between these two viruses and the other subtypes of influenza virus in human and in swine were determined by cross-HI test using hyper immune rabbit sera raised against A/Guangdong/501/2010/H1N1pdm09 and A/swine/Guangdong/L6/09. Human H1N1pdm09 antiserum showed cross-reactivity against CS H1N1 virus, with HI titer of 40; whereas antiserum against CS H1N1 virus did not cross-react with human H1N1pdm09 virus (Table 2). We observed low cross-reaction between H1N1pdm09 virus and the EA H1N1 virus, and between CS H1N1 and two SIVs (EA-like H1N2, SIV H3N2). No cross-reactivity was detected by testing CS H1N1 and H1N1pdm09 antiserum against human H1N1 seasonal virus.

### Haemagglutination inhibition assay for influenza virus antigens

Haemagglutination inhibition (HI) assay was performed for all serum samples using viral antigens of CS H1N1 and human H1N1pdm09 virus. The overall seroprevalence of anti-CS H1N1 antibodies was 6.0% (73/1214), which was much lower than that of anti-H1N1pdm09 antibodies (9.6%, 117/1214, P = 0.001 by chi-square test). The proportions of seropositive participants for each age group, exposed status (exposed and control), work type and sex were calculated according to the binomial distribution and listed in Table 3. After adjusting for sex and age, the unconditional logistic model revealed that occupational exposure status was significantly associated with the prevalence of CS H1N1 seroreactivity, as shown in Table 4. Seropositive rates for CS H1N1 were 9.3% (66/712) in exposed group and 1.4% (7/502) in unexposed group. Participants who reported occupational exposure to pigs are at a significantly higher risk for CS H1N1 infections (adjusted OR 7.23, 95% CI 3.29-15.88, P < 0.01) than those who did not occupationally expose to pigs. Similarly, after controlling for sex and exposure status, age group of 26–50 years was found to be significantly associated with CS HINI serologic outcomes. The seropositive rates for participants ≤25 years, 26–50 years and ≥51 years were 2.1% (5/83), 8.0% (62/506) and 6% (6/123) respectively. Variables including sex and work type were also tested; however, none of these had a significant association with CS H1N1 seroprevalence in unconditional logistic model.

While for H1N1pdm09 virus, the unconditional logistic regression analysis revealed no significant association between seroprevalence and occupational exposure status. Seropositive rates were 8.4% (60/712) and 11.4% (57/502) in exposed and unexposed group respectively. Variables including sex and work type didn’t affect the outcome (Table 4). A significant association between age and seropositive rate to H1N1pdm09 virus was identified after controlling for confounders (P < 0.01). Among those less than 25 years old, the prevalence of seroprotection was comparably high at about 19.8% (48/243). Seroprotection was 7.6% (59/778) among those aged 26–50 and 5.2% (10/193) among those ≥51 years old. These results suggest that the H1N1pdm09 virus infection is prevalent among young adults.

The overall geometric mean titers (GMT) both for CS H1N1 and H1N1pdm09 virus exhibited skewed distribution with a strong shift to the left, since a high proportion of sera yielded HI titer < 40. The GMT value of the exposed group and non-exposed group were 21.8 (95% CI 21.3–22.2) and 20.2 (95% CI 20.0–20.4) for CS H1N1, 21.7 (95% CI 21.3–32.0) and 22.6 (95% CI 21.9–23.4) for H1N1pdm09 virus (Table 5). Significant difference in the CS H1N1 titers between different age groups (P < 0.01) was observed. The GMTs of serum samples from those aged 26–50 years old were significantly higher (p < 0.01) than the GMTs of the samples from others. In contrast, for H1N1pdm09 virus, comparable high GMT was observed for those less than 25 years old (P < 0.01), with a GMT value of 24.7 (95% CI 23.3–26.2). And GMT values for those aged 26–50 and those more than 51 years old were 21.5 (95% CI 21.1–22.0) and 21.3 (95% CI 20.5–22.1) respectively.

Neutralization test was also conducted for 63 randomly selected serum samples in our study (data not shown). 88.9% and 85.7% agreement were obtained between the results of HI and NT tests for CS H1N1 and H1N1pdm09 virus respectively. 95.7% serum samples that were NT positive were also HI positive.

## Discussion

Zoonotic infections with swine influenza viruses have been documented frequently^21^,^22^. However the SIV associated human infections may not be fully quantify by these literatures, since the majority related case reports were based on virological diagnosis of clinical samples, and most zoonotic influenza transmissions from swine to humans may not be diagnosed^23^. Seroepidemiology studies which can evaluate general risk posed by swine influenza are needed^24^. Guangdong province, located in southern China, has been one of the putative influenza epicenters, owing to its large swine production industry. Various genotypes of influenza viruses have been isolated from pigs in this area^13^. Swine H1N1 viruses including EA H1N1, CS H1N1 and H1N1pdm09 virus are co-circulating in the sampled pig population^25^. Previous study revealed that 11.7% of swine farm residents in Southern China had sero-antibodies against EA SIV^17^. In the present study, seroprevelance against CS H1N1 virus and H1N1pdm09 virus among swine workers in Guangdong province, China, was assessed and the general risk of infection was evaluated.

Our study provides data for the first time on the prevalence of antibodies against CS H1N1 virus in swine workers in Guangdong province. Compared to the non-exposed control group, a significantly higher proportion of subjects with occupational contact to pigs exhibited positive seroreaction against the CS H1N1 virus. This was consistent with a previous serologic study conducted also in southern China, showing that occupational exposure of swine farm residents and veterinarians to pigs may increase their risk of infection with EA H1N1 and occupational exposure to swine is an important factor in zoonotic influenza transmissions from swine to human^17^. In this study, we also specifically associated occupational diversity to seropositivity to swine influenza virus. Work type was proved not a factor of significance. Similar seroprevalence was identified among different work type groups including retailers of goods other than pork in food market. This group of participants was enrolled and regarded as occupational exposure populations in our study. The undifferentiated seroprevalence to SIV suggest that the retailers of goods other than pork in food market are also at markedly increased risk for swine influenza virus infections. They may acquire SIV infections through direct exposure to swine or through human to human transmission, although laboratory based evidence for the latter explanation is spare. The crowded stalls and dense population on wet market in Guangdong province facilitates close contact between pigs and human and between human and human, thus increasing the likelihood of interspecies transmission. Other factors including age and sex of swine workers regarding SIV infections were assessed. Participants aged 26–50 years were at a high risk of CS H1N1 infections. This was in contrast to previous study conducted by Christopher W *et al*. in the USA^26^. In that study, being ≥50 years of age was associated with SIV seropositivity. Two reasons may explain this difference: Firstly, the genotype and evolution of swine H1N1 influenza viruses differ vastly in China and the USA. Isolation of CS H1N1 virus in the USA was in 1930s, while common infection of this virus in China was since 1996. Secondly, the effect of age and exposure over time to swine is positively related. Compared to swine farm owners aged ≥50 years in the USA, most of swine workers in China were young or mid- aged.

We didn’t observe significant different seropositive rates for 2009 pandemic H1N1 virus between swine workers and controls. It is documented that mortality rates and transmissibility for H1N1pdm09 virus are lower than those for previous influenza pandemics. And the 2009 pandemic virus is now circulating in the human population similarly to a seasonal human influenza virus^27^. Although transmission of 2009 pandemic H1N1 virus from humans to pigs occurred shortly after its emergence, neither complete H1N1pdm09 viruses nor their surface genes have established in Chinese pigs^28^. The main persistent H1N1pdm09 virus-origin reassortant forms only had their internal genes come from H1N1pdm09 virus, and their surface genes were primarily of European avian-like (EA) or human H3N2-like SIV origin. Exposure to swine is a less important consideration for H1N1pdm09 virus infection than for CS H1N1 virus infection in China. This was in contrast to previous serologic surveys conducted in other countries^29^. In Germany, increased risk of infections with 2009 pandemic H1N1 viruses were identified for occupational exposure populations^18^. The high proportion of HI antibodies in German swine workers may be explained by the prevalence of this virus and its reassortants in the German pig population^30^,^31^,^32^. Besides, the overall percentage of reactive sera against H1N1pdm09 virus in our study was lower than that of Germany. A previous meta-analysis of H1N1pdm09 virus serological studies from 19 countries showed significant differences in prevalence by region^33^. Results presented in our study are partially in line with data previously raised in Guangdong province in 2010, showing a seroprevalence of 14.3% for H1N1pdm09 virus among non-vaccinated subjects^34^. In addition, significant association between age and seropositivity was identified for H1N1pdm09 virus. The major impact of age was apparent for younger populations. For age group less than 25 years, the prevalence of seroprotection was comparably higher than for other age groups. Similar age distribution was observed in Europe^30^,^35^, the USA^36^ and Canada^37^. And the age-related pattern of potential susceptibility should be taken into account in the process of influenza control strategies making.

The present study has several limitations. It has been regarded that there exists obvious antigenic similarity between 2009 pandemic H1N1 virus and contemporary CS H1N1 virus^29^. In our study, low cross-reactivity was observed by testing H1N1pdm09 antiserum against CS H1N1 virus but not by testing CS H1N1 antiserum against H1N1pdm09 virus. Much of this discrepancy is to do with low antibody titer of the control CS H1N1 antiserum (HI 320) used in our study. The 25 positive samples showing reactivity towards both tested virus in our study may reflect a certain level of cross-reactivity and complicated the interpretation of HI test results. Currently, all main SIV subtypes are co-circulating in China. While, only sero-antibodies against 2009 pandemic H1N1 virus and CS H1N1 virus were evaluated in our study. To comprehensively understand the risk of zoonotic influenza transmission and to rule out cross-reactivity against other viruses circulating in the Chinese swine population and in human population, virological and serological studies included adequate strains are in needed. Besides, NT assay was performed only for partial serum samples and was not included in data processing in our study. Compared to HI, the NT assay may be more sensitive to detect low levels of sero-antibodies. The overall sero-antibodies against these two viruses may have been underestimated.

In conclusion, our present study has documented evidence for swine influenza virus infection in persons whose professions involve contact with swine. Compared to none exposure groups, workers with occupational swine exposure showed significant higher seroprevalence to CS H1N1 virus, a representative swine viral strain in Guangdong province. For 2009 pandemic H1N1 virus, similar seroprevalence was identified for exposed group and control group, due to its low probability in swine populations. The observed difference in seroreactivities for these two subtypes emphasize the necessity of regular surveillance both in pigs and humans. Recommendations for pandemic preparedness need to be adjusted accordingly to take into account virus subtype and antigenicity changes.

## Additional Information

**How to cite this article**: Wu, J. *et al*. Anti-Human H1N1pdm09 and swine H1N1 Virus Antibodies among Swine Workers in Guangdong Province, China. *Sci. Rep*. **5**, 12507; doi: 10.1038/srep12507 (2015).

## Figures and Tables

**Table 1:** Baseline characteristics of study participants.

Characteristic	Swine workers	Controls
	no. (%), n = 712	no. (%), n = 502
Sex		
M	446 (62.6)	299 (59.6)
F	266 (37.4)	203 (40.4)
Age group		
≤25 years	83 (11.7)#	160 (31.9)
26–50 years	506 (71.1)	272 (54.2)
≥51 years	123 (17.3)	70 (13.9)
Profession		
Swine keepers	126 (17.7)	NA*
Pork processer	169 (23.7)	NA
Retailers of goods other than pork	360 (50.6)	NA
Quarantine officials	57 (8)	NA

^#^Significantly different than controls at α = 0.05.

^*^NA, not applicable.

**Table 2:** Hemagglutination-inhibition titers of control sera to reference virus strains used in this study.

Reference influenza viruses	Control serum	
	A/Guangdong/501/2010/H1N1pdm09 rabbit antiserum	A/swine/Guangdong/L6/09 rabbit antiserum
A/Guangdong/501/2010(pdmH1N1)	640	<10
A/Sichuan/SWL1/2009(pdmH1N1)	640	<10
A/Tianjin/Jinnan/H1N1(seasonal H1N1)	<10	<10
A/swine/Guangdong/L6/09(CS H1N1)	40	320
A/swine/Guangdong/SS1/2012(H1N1)(EA H1N1)	40	<10
A/swine/Guangdong/3/2013(H1N2)(EA-like)	<10	40
A/swine/Guangdong/4/2009(H3N2)	<10	40
A/swine/Guangdong/1/08(H4)	<10	<10
A/swine/Guangdong/1/10(H9)	<10	<10
A/Fujian/Tongan/196/2009(H3N2)	<10	<10
B/Chongqin/Yuzhong/1361/2013(By)	<10	<10
B/Beijing/Haidian/1386/2013(Bv)	<10	<10

**Table 3:** Seroprevalences of antibodies against 2 influenza viruses in Guangdong, China.

Characteristic	Classic swine H1N1 virus		2009 pandemic H1N1 virus	
	Swine workers	Control	Swine workers	Control
	n = 712	n = 502	n = 712	n = 502
Sex				
M	47 (10.5%)*	5 (1.7%)	35 (11.7%)	22 (10.8%)
F	19 (7.1%)	2 (1.0%)	37 (8.3%)	23 (8.6%)
Age group				
≤25 years	2 (2.4%)	3 (1.9%)	18 (21.7%)	30 (18.8%)
26–50 years	58 (11.5%)	4 (1.5%)	40 (7.9%)	19 (7.0%)
≥51 years	6 (4.9%)	0 (0.0%)	2 (1.6%)	8 (11.4%)
Work type				—
Swine keepers	10 (7.9%)	—	9 (7.1%)	—
Pork processer	17 (10.1%)	—	18 (10.7%)	—
Retailers of goods other than pork	36 (10.0%)	—	29 (8.1%)	—
Quarantine officials	3 (5.3%)	—	4 (7.0%)	—

^*^Data are no. (%) of subjects.

**Table 4:** ORs for increased serologic response against 2 influenza viruses, determined by logistic regression modeling.

Characteristic	Classic swine H1N1 virus			2009 pandemic H1N1 virus		
	no. (%)	Unadjusted OR (95% CI)*	Adjusted OR (95% CI)	no. (%)	Unadjusted OR (95% CI)(95% CI)*	Adjusted OR (95% CI)
Sex						
M	52 (7.0)	0.63 (0.37–1.05)	—	72 (9.7)	0.99 (0.67–1.47)	—
F	21 (4.5)	Ref	—	45 (9.6)	Ref	—
Age group						
≤25 years	5 (2.1)	0.66 (0.20–2.18)	1.03 (0.30–3.47)	48 (19.8)	4.50 (2.21–9.17)#	4.44 (2.16–9.13)#
26–50 years	62 (8.0)	2.70 (1.15–6.33)#	2.70 (1.15–6.38)#	59 (7.6)	1.50 (0.75–2.99)	1.50 (0.75–3.00)
≥51 years	6 (3.1)	Ref	Ref	10 (5.2)	Ref	Ref
Participant property						
Exposed	66 (9.3)	7.23 (3.29–15.88)#	6.36 (2.87–14.10)#	60 (8.4)	0.72 (0.49–1.05)	0.95 (0.64–1.43)
None exposed	7 (1.4)	Ref	Ref	57 (11.4)	Ref	Ref
Work type						—
Swine keepers	10 (7.9)	0.78 (0.37–1.61)	—	9 (7.1)	0.88 (0.40–1.91)	—
Pork processer	17 (10.1)	1.00 (0.55–1.85)	—	18 (10.7)	1.36 (0.73–2.53)	—
Retailers of goods other than pork	36 (10.0)	0.50 (0.15–1.68)	—	29 (8.1)	0.86 (0.29–2.55)	—
Quarantine officials	3 (5.3)	Ref	—	4 (7.0)	Ref	—

^*^OR, odds ratio; CI, confidence interval.

^#^Values are statistically significant.

**Table 5:** Geometric mean titers of antibodies against 2 influenza viruses in Guangdong, China.

Characteristic	Classic swine H1N1 virus, GMT (95% CI)				2009 pandemic H1N1 virus, GMT (95% CI)			
	Swine workers	Control	Total		Swine workers	Control	Total	
	n = 712	n = 502	1214	*P value	n = 712	n = 502	1214	P value
Sex				0.069				0.969
M	22.1 (21.5–22.8)#	20.3 (20.0–20.5)	21.4 (21.0–21.8)		21.6 (21.0–22.1)	22.7 (21.7–23.6)	21.2 (21.5–22.5)	
F	21.2 (20.7–21.8)#	20.1 (19.9–20.3)	20.8 (20.4–21.2)		21.7 (21.3–22.2)	22.6 (21.5–23.8)	22.2 (21.5–22.9)	
Age group				<0.001				<0.001
≤25 years	20.5 (19.8–21.3)	20.4 (19.9–20.8)	20.4 (20.0–20.8)		25.1 (22.7–27.7)	24.5 (22.9–26.3)	24.7 (23.3–26.2)	
26–50 years	22.2 (21.6–22.8)#	20.2 (20.0–20.4)	21.5 (21.5–22.0)		21.5 (21.0–22.0)	21.6 (20.8–22.4)	21.5 (21.1–22.0)	
≥51 years	20.9 (20.1–21.7)	20.0 (20.0–20.0)	20.6 (20.1–21.1)		20.5 (19.9–21.1)#	22.7 (20.8–24.9)	21.3 (20.5–22.1)	
Work type				0.69		—		0.658
Swine keepers	20.6 (20.6–22.6)	—	21.6 (20.6–22.6)		21.4 (20.4–22.3)	—	21.4 (20.4–22.3)	
Pork processer	20.2 (21.1–23.4)	—	22.2 (21.1–23.4)		22.2 (21.2–23.4)	—	22.2 (21.2–23.4)	
Retailers of goods other than pork	21.8 (21.2–22.4)	—	21.8 (21.2–22.4)		21.6 (21.0–22.2)	—	21.6 (21.0–22.2)	
Quarantine officials	21.0 (19.8–22.3)	—	21.0 (19.8–22.3)		21.5 (20.0–23.2)	—	21.5 (20.0–23.2)	
Total	21.8 (21.3–22.2)#	20.2 (20.0–20.4)	—		21.7 (21.3–22.2)	22.6 (21.9–23.4)	—	

^#^Values are statistically significant when compared with those of control.

^*^P value: Value is calculated among each category for total cases.
